# A Scalable and Extensible Logical Data Model of Electronic Health Record Audit Logs for Temporal Data Mining (RNteract): Model Conceptualization and Formulation

**DOI:** 10.2196/55793

**Published:** 2024-06-24

**Authors:** Victoria L Tiase, Katherine A Sward, Julio C Facelli

**Affiliations:** 1 Department of Biomedical Informatics University of Utah Salt Lake City, UT United States; 2 College of Nursing University of Utah Salt Lake City, UT United States

**Keywords:** burnout, professional, nursing, nurse, electronic health record, EHR, data modeling, data set, temporal machine learning, machine learning, ML, artificial intelligence, AI, algorithm, predictive model, predictive analytics, practical model

## Abstract

**Background:**

Increased workload, including workload related to electronic health record (EHR) documentation, is reported as a main contributor to nurse burnout and adversely affects patient safety and nurse satisfaction. Traditional methods for workload analysis are either administrative measures (such as the nurse-patient ratio) that do not represent actual nursing care or are subjective and limited to snapshots of care (eg, time-motion studies). Observing care and testing workflow changes in real time can be obstructive to clinical care. An examination of EHR interactions using EHR audit logs could provide a scalable, unobtrusive way to quantify the nursing workload, at least to the extent that nursing work is represented in EHR documentation. EHR audit logs are extremely complex; however, simple analytical methods cannot discover complex temporal patterns, requiring use of state-of-the-art temporal data-mining approaches. To effectively use these approaches, it is necessary to structure the raw audit logs into a consistent and scalable logical data model that can be consumed by machine learning (ML) algorithms.

**Objective:**

We aimed to conceptualize a logical data model for nurse-EHR interactions that would support the future development of temporal ML models based on EHR audit log data.

**Methods:**

We conducted a preliminary review of EHR audit logs to understand the types of nursing-specific data captured. Using concepts derived from the literature and our previous experience studying temporal patterns in biomedical data, we formulated a logical data model that can describe nurse-EHR interactions, the nurse-intrinsic and situational characteristics that may influence those interactions, and outcomes of relevance to the nursing workload in a scalable and extensible manner.

**Results:**

We describe the data structure and concepts from EHR audit log data associated with nursing workload as a logical data model named RNteract. We conceptually demonstrate how using this logical data model could support temporal unsupervised ML and state-of-the-art artificial intelligence (AI) methods for predictive modeling.

**Conclusions:**

The RNteract logical data model appears capable of supporting a variety of AI-based systems and should be generalizable to any type of EHR system or health care setting. Quantitatively identifying and analyzing temporal patterns of nurse-EHR interactions is foundational for developing interventions that support the nursing documentation workload and address nurse burnout.

## Introduction

### Workload as a Contributor to Burnout

Nursing workload, representing the amount of time, physical, and cognitive effort needed to provide nursing care, is an ongoing concern in health care settings and can pose significant challenges for nurses [1]. Increasing cost pressures and the lingering effects of COVID-19 have resulted in nurses taking care of sicker patients than in the past [2]. Many health care facilities experience a shortage of qualified nursing staff, resulting from factors such as insufficient recruitment and retention efforts or high turnover rates due to burnout [2]. There is also a higher demand for nurses given the aging population so that supply no longer meets demand [2]. As more nurses continue to exit the workforce, the workload increases further for those who remain on the job [3].

### Measuring Nursing Workload

Historically, the most commonly used measure of nursing workload is the nurse-patient ratio [4], referring to the number of patients assigned to a nurse during a specific shift or time frame [5]. These ratios play a role in determining the amount of direct patient care that each nurse must provide, which directly impacts workload and the quality of care nurses can deliver. Research on patient ratios provides evidence connecting nurse staffing to patient outcomes and more specifically to patient safety [4]. However, establishing a safe and effective nurse-patient ratio is an ongoing challenge for health care organizations and policy makers [6].

A major weakness to estimating nursing workload at this macro level is that this approach does not account for differences in patient illness or the amount of care needed by an individual patient, nor does it account for contextual and organizational characteristics that impact workload [3]. Additionally, recent studies found evidence that not all nurses, even within the same hospital unit, practice the same way; therefore, examining individual nurse practices becomes important in examining workload [4]. In addition to nurse-patient ratios, nursing workload has also been studied as a qualitative experience and estimated via time-motion studies, whereas the subjective and intrusive nature of these approaches suggests that more research is needed from a quantitative or mixed methods perspective [7,8].

### Electronic Health Record Data as an Estimate of Workload

The health care industry has access to a massive amount of data that can be analyzed for trends, patterns, and insights [9]. Data regarding clinical care are encompassed within the electronic health record (EHR). Researchers have leveraged EHR interactions to track clinical work activities and associated workload for physicians, and EHR interactions or use patterns were used to predict physician departures and burnout [9-11]. Nursing clinical care and documentation are distinct from physician care; however, to date, nursing workload has not been similarly evaluated from EHR data. Although it is well-established that nursing work extends beyond what is recorded, we posit that EHR documentation could be a reasonable proxy for nursing workload.

As part of regulatory requirements, health care organizations must record and track EHR activity at the user level, including log-on attempts, what patient records are accessed, what documentation was entered, and the date and time of access [12]. These user-level metadata, stored as EHR audit logs, are an untapped resource that have potential to provide clinical insights [13]. Some nurse researchers have explored the suitability of EHR audit logs to understand the documentation burden [14]. However, these metadata are complex, change over time, and varied in how they are aggregated into higher-level measures [15]. To effectively use these data, it is necessary to structure the raw audit logs as part of a consistent and scalable logical data model that can be consumed by artificial intelligence (AI) algorithms.

### Logical Data Models

A logical data model is a set of specifications that identifies the primary data concepts and relationships between them, serving as a blueprint or template on how information is organized for analysis [16]. Machine learning (ML) models typically organize data as vectors. In this context, a vector is a specific way to represent data as a matrix of values. In planning for ML, a logical data model can define the main categories of vectors that serve as model inputs or outputs, which can be implemented as a physical model consumable by any ML algorithm [16]. A logical data model is intentionally abstract, not constrained by either the data sources or the actual structure that will store the data [16]. Ultimately, the aspects of the logical data model will inform the physical implementation of the model [17].

### Objective

We posit that gathering and modeling sufficient data that are reliable, reproducible, and generalizable, and that represent nursing contributions within the context of work activities and workload are achievable with data science–based research. We hypothesize that assessing the nursing workload requires objective measurement and a standardization of data elements that represent clinical activities and other nursing workload influences, which can be used as targets for modeling interventions at scale. This hypothesis is supported by the findings from a systematic review of studies using EHR audit logs to observe clinical activities [10]. Because EHR audit logs record all types of interactions with the health record, the logs may offer insights into how nurses interact with the EHR and the extent to which EHR interactions reflect workflow and workload. Our objective was to conceptualize a logical data model based on EHR audit log data as a first step toward analysis of nursing EHR documentation workflows. This is somewhat analogous to developing a conceptual framework before starting a traditional analysis.

Using concepts derived from our previous work in studying temporal biomedical data patterns [18,19], we formulated a data structure that can describe nurse-EHR interactions, nurse-intrinsic and situational characteristics, and nurse outcomes of interest in a scalable and extensible manner. We believe the selected features will allow for metadata aggregation into EHR use measures that can be used for a variety of nurse-centric outcomes. We then conceptually instantiated the model with an analysis plan for a quantitative study of the characteristics and expected outcomes associated with nurse-EHR interactions using AI and temporal ML methods. Although our purpose is to focus on the EHR audit log data, we also provide details on additional data sources that will be needed to instantiate the logical data model. We conceptualize how the model could be used to support data science methods based on AI and ML approaches. The physical implementation of the model will be described in subsequent work.

## Methods

### Study Design

To develop this proposed conceptual framework, we began with a search of the literature, and expanded upon previous work that examined the workload of physicians using EHR data and researched components of nursing workload that can be extracted from other (non-EHR) health system databases [20]. Two experienced nurses iteratively refined the concepts and interactions until consensus was achieved. We conducted a preliminary review of the audit log from a commercial EHR vendor (Epic) implemented at an academic medical center to confirm that the conceptualized model corresponded to generalizable structures for audit log data.

### Ethical Considerations

Given that EHR audit log data are user-centric and not patient-centric, we protected user identity by deidentifying users and aggregating activity according to the generic user role “nurse.” After consultation with the University of Utah Institutional Review Board, the protocol received an exemption determination.

### EHR Audit Log Metadata

In our review of the EHR audit logs, we found that nursing interactions with the EHR extend beyond clinical data input. Within the EHR audit logs, we were able to distinguish between data review and data input, identify the particular section of the EHR accessed (eg, medication administration record, best-practice advisories, or notes), and determine the workflow activities such as navigation between records. We focused on data specific to nurse users. In their current form, these data lack a hierarchical data structure and do not contain a taxonomy related to specific user or task types.

## Results

### Data Model: Concepts

The data model, which we named RNteract, contains elements that describe the nurse tasks (NTask), nurse characteristics (NType), specific type of patient or *patient panel* (NPanel), and the resulting nurse-relevant outcomes (NOutcome). Each concept will be represented via a value set for physical implementation of the model. The value sets can be defined based on the purpose or goals of the analysis. As an example, the NTask value set can describe a general type of task (such as *medication tasks*) or can be more granular (eg, specific steps of medication administration) depending on the objectives of the model.

### Model Structure: Vectors

This logical data model is intended to support temporal ML models. These approaches are grounded in the concept of vectors or numerical arrays. A vector is a mathematically based approach for expressing and organizing data in a predefined manner. Vectors in ML represent input data, including bias or weights. In the same way, output from an ML model can be represented as a vector. To perform a given ML task, the first step is to represent the input. For this logical data model, we provide a set of specifications and primary data structures with a focus on the NTask input.

### Model Component: NTask

Our initial focus will be on defining the NTask element. For each nurse, ID=k, we define a vector <NTask(k, i)>, i=1 to N, as a vector of dimension N, where N is the number of time periods considered in the study. Any resolution (eg, day, hour, 30-minute intervals, each minute) can be used for the time periods. The individual nurse performing the task is represented by k and the i component of the vector corresponds to the time interval under study.

The value representing the specific task is taken from a value set that describes the nursing tasks considered in the model and can be as general or specific as desired. For instance, if a value set for tasks has been defined broadly such that 1=no EHR interaction, 2=read EHR data, and 3=input EHR data, if the nurse identified with ID 55 has been reading the EHR for the first time period, does nothing with the EHR for the next three periods, and then writes a note in the EHR for the fifth time period, the corresponding NTask vector will be (55, 2, 1, 1, 1, 3).

The NTask vector will allow the classification of activity patterns in EHR data for any finite number of nurses, for an arbitrary set of tasks, and for any finite time resolution. Instantiation of the model would result in a set of vectors that can then be classified with temporal ML methods to model nurse activity trajectories and other patterns of nurse-EHR interactions. Resulting nurse activity trajectories can be associated with quantitative descriptors of NType, NPanel, and NOutcome, which are defined in further detail below.

### Additional Components

#### NType

NType describes the nurse user within the EHR audit log data. For each nurse with ID=k, the NType data element is a vector of dimension M in which the i component of the vector <NType (k, i)>, i=1 to M, is an integer, real, or categorical value defined by a value set that describes both intrinsic and situational characteristics of the nurse. The dimension of the vector, M, is defined by the number of characteristics we desire to model and to assign to a nurse.

Audit logs, by themselves, have only limited information about the nurse characteristics other than identification of individual users. From the EHR audit logs, we can determine the location of where the EHR interaction occurred (inferred from the computer location), and consequently can infer additional information based on the most common location of documentation and characteristics such as the environment the nurse primarily practices in (eg, medical/surgical environment or an intensive care unit). However, we will need to access data from other sources to incorporate other nurse characteristics. These data may be available from the EHR scheduling system or other auxiliary systems such as credentialing systems or human resources databases. Examples of a desired model value set using these additional data sources may be:

1. Nurse employment length, integer (eg, number of years in the job, workplace).

2. Nurse’s highest professional accreditation, categorical variable (eg, licensed practical nurse, registered nurse [RN], nurse practitioner).

3. Average number of hours worked per week, integer, which is potentially derivable from the EHR audit log (eg, based on first and last EHR interactions in a 24-hour period).

4. Nurse primary assignment, categorical variable (eg, operating room [OR], intensive care, medical/surgical). This information can potentially be inferred from the EHR audit log based on the primary location of the computer used for documentation.

As an example, if a nurse with ID 55 has been in the organization for 5 years, is an RN, worked 48 hours per week, and is primarily assigned to the OR, the corresponding vector would be NType=(55, 5, RN, 3, OR).

#### NPanel

NPanel is a vector to describe the patient context for the tasks. The data element for the patient panel assigned to nurse with ID=k is a vector of dimension O in which the i element of <NPanel (k, i), i=1 to O, is an integer, real, or categorical value defined by a value set that describes the characteristics and the acuity of the patients in the panel. The dimension of the vector, O, is defined by the number of features used to describe the patient panel. For instance, this data element may accommodate the complexity level of the patients or the average length of stay of the patients. These data will not be available directly from EHR audit logs, but may be obtained from the admission, discharge, and transfer messages; a financial system; patient acuity; or other clinical sources. An example of a possible value set for a patient panel might be:

1. Admission from the emergency room; integer, number of patients admitted.

2. Admission from the OR; integer, number of patients admitted.

3. Discharged to home; integer, number of patients discharged.

4. Average length of stay; integer.

An example for this component is if nurse ID=55 has a panel of 12 patients with 1 admission from the OR, 2 discharges, and an average length of stay of 4, the corresponding vector would be NPanel=(55, 0, 1, 2, 4).

#### NOutcome

The NOutcome element for a nurse with ID=k is a vector of dimension P in which the i element of <NOutcome (k, i)>, i=1 to P, is an integer, real, or categorical value defined by a value set that describes long-term changes in the status of the nurse and P is the number of features included in the model to describe the nurse outcomes. This data element can accommodate events such as resignation, promotion, or salary increase. Other potential outcomes may include use of time off, patient safety events, and preventable harm. As with NType, these data may be available from the EHR or other ancillary systems. Examples of a value set may be:

1. Nurse salary increase; Boolean: YES, NO.

2. Nurse resignation; Boolean: YES, NO.

3. Nurse promotion; Boolean: YES, NO.

4. Nurse missed work; integer, number of days that the nurse missed work.

For example, if nurse ID=55 did not receive a salary increase, did not resign, was not promoted, but missed 6 days in the last month, the corresponding vector would be NOutcome=(55, NO, NO, NO, 6).

Such long-term outcomes will not be recorded in EHR audit logs and must be obtained from other data sources. However, audit logs may hint at intermediate outcomes and workflows, including workflows such as charting in a block of time (perhaps charting after the shift) versus charting throughout the shift. These intermediate outcomes may suggest hypotheses for further exploration, such as the extent to which charting in a block of time may reflect a high workload.

### Extensibility/Generalizability

The definitions of NType, NPanel, and NOutcome are extensible and can accommodate any finite number of properties associated with any nurse. While we recognize that these properties may change over time, we chose to make NType, NPanel, and NOutcome independent of time because the variations in these categories are usually much slower than the variations of the time-dependent NTask vector. If necessary, categorical variables can be transformed into integers by defining a table transformation. This makes NType, NPanel, and NOutcome amenable to any ML approach that may be considered.

### Conceptual Instantiation for AI and ML

Using temporal unsupervised classification [21-23], the <NTask (i, k)> nurse-EHR temporal interaction patterns can be classified through pattern recognition. The resulting archetypical activity patterns or clusters can be associated with any of the properties described in either NType, NPanel, or NOutcome, where the clusters can be described as enriched or depleted of any of the properties coded by these data elements. Using traditional statistics, the significance of the enrichments/depletions can be estimated.

**Figure 1:**
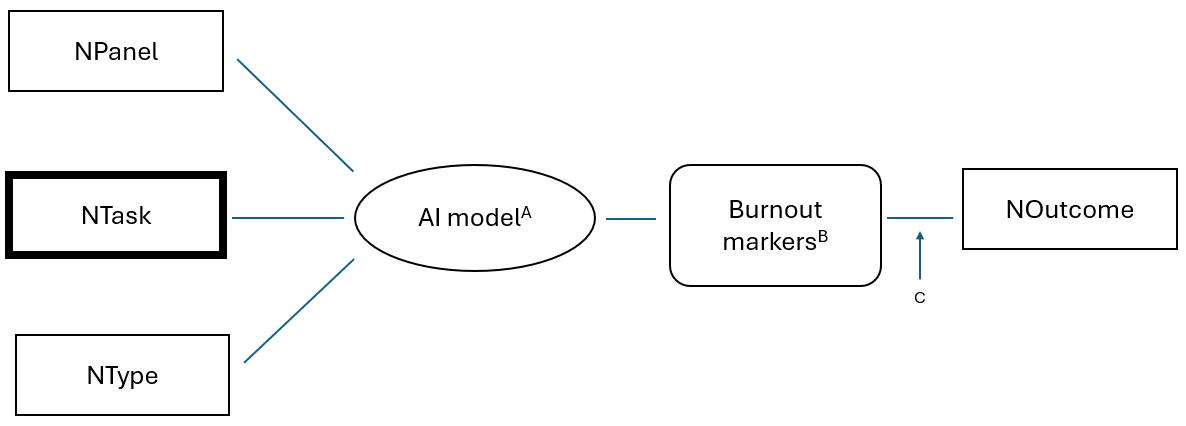
RNteract logical data model. A: Any state-of-the-art AI model can be accommodated; B: electronic health record–derived time patterns for charting, navigation, or searching; C: allows for the inclusion of other contributors to burnout. AI: artificial intelligence; NOutcome: nurse-relevant observations or outcomes; NPanel: patient panel being cared for; NTask: nurse tasks; NType: nurse characteristics.

Descriptive analysis of the NTask clusters can be used to gain insights from interaction patterns, which will allow for the development of predictive models for each cluster associated with the inputs for NTask, NType, and NPanel descriptors and outputs of interest described by NOutcome. As shown in Figure 1, this model allows for predictive modeling and is not constrained by the selection of AI method. This intersection of nurse types, patient panels, and tasks, aggregated over time and across patient encounters, can be analyzed using a range of state-of-the-art AI techniques for different purposes, revealing burnout markers such as charting, navigation, and searching patterns. Although we recognize that burnout is not entirely measurable from EHR interactions, we assumed a set of burnout markers with concrete measurements that can be obtained from the user-centric logs. The logical data model also allows for the inclusion of other contributors to burnout that can be incorporated prior to describing the outcome.

## Discussion

### Clinical Implications

This paper describes the development of a scalable and extensible logical data model to represent interactions between nurses and EHR systems as recorded in EHR audit logs. We identified a general methodology for identifying concepts related to nursing workload and applied this methodology to EHR interactions as reflected in EHR audit logs.

AI-based systems have increasingly been incorporated into health care [24]. To date, research on AI in health care has largely neglected to consider real-world scenarios or real-world effect on outcomes [25]. Uses of AI in nursing span from virtual assistants to patient monitoring and predictive modeling [26]. AI can also contribute to nursing by helping to streamline workflows and analyze vast amounts of data for evidence-based recommendations, helping nurses make more informed decisions about patient care [26-28]. Nurses have had only limited engagement in workflow or workload modeling efforts, despite the potential for AI-based systems to contribute to advanced, effective, efficient care, such as through more effective ways to access and organize information from EHRs [25].

The metadata in audit logs can be complex and unstructured, making them difficult to interpret. Advanced analytics such as ML and data mining are necessary to identify patterns and insights in such complex data; however, to use these methods effectively, the data need to be structured in a way that it is consumable by AI and ML applications.

The logical data model presented herein provides a structured framework for organizing the vast amount of data generated in audit logs. This model can help in categorizing data elements and defining their relationships with a flow of logical reasoning, which is essential for understanding and using the data effectively. With a well-defined logical data model, it becomes easier to analyze audit log data by providing a clear representation of data flows and structures. We believe that this data model will provide a better understanding of nursing-related data elements and will assist others in using EHR audit log data to effectively model nursing workload or related outcomes of interest.

Prior to a physical implementation of the model (details will be described elsewhere), the non-EHR audit log data sources may require additional representation. However, as many of the other data sets have been described in the literature, our focus is to promote the broader use of EHR audit log data to understand the nursing workload [29,30].

### Limitations

The data model was developed based on the literature and on the audit log of a single organization’s EHR. The audit logs of other organizations using the same or different EHR systems may suggest additions or modifications to the model. Consultation with the nursing research community may lead to further refinements of the model; to this end, we envision subjecting the model to an open discussion in relevant research venues.

### Conclusion

Using concepts derived from our previous experience in studying temporal biomedical data patterns, we formulated a data model that can be used to describe nurse-EHR interactions, intrinsic and situational characteristics of nurses and patient panels, and nurse outcomes of interest in a scalable and extensible manner. The definitions of NTask, NType, NPanel, and NOutcome are extensible and can accommodate any finite number of properties associated with a nurse. Through the use of the logical data model, we conceptualize how nurse-EHR interactions could be studied using temporal unsupervised ML as well as any state-of-the-art AI methods. Future work may include extensions or modifications of the model as we test its applicability to different organizations and different EHR systems.

## Abbreviations

AI: artificial intelligence
EHR: electronic health record
ML: machine learning
NOutcome: nurse-relevant observations or outcomes
NPanel: patient panel being cared for
NTask: nurse tasks
NType: nurse characteristics
OR: operating room
RN: registered nurse
